# Clinical meaningfulness of anti‐amyloid therapies in early Alzheimer's disease: Perspectives from the East and Southeast Asia region

**DOI:** 10.1002/alz.71230

**Published:** 2026-02-22

**Authors:** Christopher Chen, Jae‐Hong Lee, Kee Hyung Park, Chaur‐Jong Hu, Vincent Mok, Vorapun Senanarong, Kaori Inaba, Amitabh Dash

**Affiliations:** ^1^ Memory, Aging and Cognition Center Department of Pharmacology Yong Loo Lin School of Medicine National University of Singapore Singapore Singapore; ^2^ Department of Neurology Asan Medical Center University of Ulsan College of Medicine Seoul South Korea; ^3^ Department of Neurology College of Medicine Gachon University Gil Medical Center Incheon South Korea; ^4^ Dementia Center Department of Neurology Shuang Ho Hospital College of Medicine Taipei Medical University New Taipei City Taiwan; ^5^ Lau Tat‐chuen Research Centre of Brain Degenerative Diseases in Chinese Gerald Choa Neuroscience Institute Li Ka Shing Institute of Health Sciences Lui‐Che Woo Institute of Innovative Medicine Division of Neurology Department of Medicine and Therapeutics Faculty of Medicine The Chinese University of Hong Kong Hong Kong SAR China; ^6^ Department of Medicine Faculty of Medicine Siriraj Hospital Mahidol University Bangkok Thailand; ^7^ Medical Department Eisai Co. Ltd Tokyo Japan; ^8^ Medical Department Eisai Singapore Pte Ltd Singapore Singapore

**Keywords:** Alzheimer's disease, anti‐amyloid therapies, Asia, clinical meaningfulness, communication

## Abstract

Alzheimer's disease (AD) is a neurodegenerative disease characterized by progressive accumulation of toxic amyloid species. The rising prevalence of AD in Asia has made it an increasing public health concern, placing a substantial burden on the economy and healthcare systems. Anti‐amyloid therapies (AATs) have demonstrated statistically significant slowing of disease progression at the group level in pivotal trials in early symptomatic AD. We explore the clinical meaningfulness of AATs and considerations impacting on its meaning in East and Southeast Asia. We acknowledge that there is a lack of data on Asian populations, particularly from the perspectives of patients and caregivers, highlighting the need for such evidence to facilitate the successful adoption of AATs in the region. We also propose the conceptual Connect, Align, Reframe, Explain (CARE) communication framework and practical tools to support effective communication with patients and caregivers regarding the benefits of AATs.

**Highlights:**

- Anti‐amyloid therapies (AATs) have demonstrated statistically significant group‐level effects in slowing of disease progression in early symptomatic Alzheimer's disease.
- Translating clinical trial outcomes into measures of benefit that are truly meaningful to patients and caregivers is critical for the adoption of AATs.
- Asia, with its rapidly aging societies, diverse cultural norms and heterogeneous healthcare and reimbursement systems, presents a unique perspective on the clinical meaningfulness of AATs.
- The proposed Connect, Align, Reframe, Explain (CARE) communication framework concept and practical support tools, such as goal‐setting checklist, visual aids and motivational messages, can facilitate effective communication with patients and caregivers regarding the benefit of AATs.
- Optimizing the full potential of AATs in Asia requires focused efforts on understanding patients’ and caregivers’ perspectives on treatment benefits, building regional registries to collect real‐world data, and aligning care frameworks.

## 1 INTRODUCTION

Alzheimer's disease (AD) is a progressive neurodegenerative disorder that causes significant distress for patients and their families and places a substantial burden on the economy and healthcare systems in Asia.^1^

Conventional management of AD has been limited to symptomatic treatment that does not affect the underlying pathophysiological process of AD. The advent of disease‐modifying therapies targeting Aβ species ushers in a new era in the treatment of AD, offering hope of delaying disease progression in individuals with early‐stage AD. Anti‐amyloid therapies (AATs), such as lecanemab and donanemab, have demonstrated reductions in amyloid burden and a corresponding slowing of cognitive decline in patients with early‐stage AD,^2^, ^3^ earning full approvals from the United States Food and Drug Administration (US FDA). Lecanemab preferentially binds to smaller Aβ aggregates/protofibrils present early in the course of AD, while donanemab targets Aβ plaques–both mechanisms support the rationale for early‐stage treatment.^4^ Despite the positive outcomes of AAT treatment, some view its effect on slowing of cognitive decline to be modest with uncertain long‐term durability, posing challenges to widespread clinical adoption.

Recent revisions to the diagnostic and staging criteria of AD emphasize a biology‐based definition and the growing role of blood‐based biomarkers, underscoring the importance of evaluating the clinically meaningful benefit of treatment in early symptomatic stages.^5^, ^6^ Regulatory frameworks often define “meaningful benefit” using clinical outcome assessments (COAs), such as the Clinical Dementia Rating‐Sum of Boxes (CDR‐SB). However, these tools may not fully capture domains that truly matter to individuals living with AD and their caregivers. The Alzheimer's Association's “What Matters Most” program revealed that these important domains include thought processing, communication, daily activities, mood/emotion, social life, and general independence,^7^, ^8^ highlighting preservation of independence and daily function as a marker of meaningful benefit. As such, translating these COAs into measures of clinically meaningful outcomes and effectively communicating these meaningful benefits in patient‐relevant terms play a key role in decision‐making conversations in the clinic.

Defining clinical meaningfulness is particularly important in many East and Southeast Asian settings, where rapid demographic aging, cultural norms and healthcare system characteristics may shape perceptions of the treatment benefit of AAT. Given that AD prevalence is rising fastest in this region, even a modest delay in disease progression can substantially reduce years of dependency and caregiving needs. Family‐centered caregiving and limited acceptance of institutional care make preservation of independence especially valuable. Economic pressures add urgency, as dementia care is often paid out‐of‐pocket in low‐ and middle‐income countries, whereas systems in high‐income countries require clear evidence of benefit to justify reimbursement. Genetic and epidemiological differences from Western counterparts, including lower apolipoprotein E (*APOE*) *ε4* prevalence but higher cerebrovascular risk, further underscore the need for Asia‐specific perspectives on clinically meaningful benefit. Developed by an expert panel of six neurologists across East and Southeast Asia (Hong Kong, Singapore, South Korea, Taiwan, and Thailand), this article explores the clinical meaningfulness of AATs and considerations impacting on its meaning in these regions, and proposes a conceptual framework and practical tools that can be used to support effective communication with patients and caregivers regarding the benefits of AATs.

## 2 CLINICAL BENEFITS OF AATs IN ASIAN POPULATIONS IN CLARITY AD AND TRAILBLAZER‐ALZ 2 TRIALS

Lecanemab became the first AAT to receive standard approval by the US FDA in July 2023,^9^ and has been approved in several Asian countries, including China, Hong Kong, Japan, South Korea, Singapore, Taiwan, and Thailand.^10^ Donanemab was approved later by the US FDA in July 2024,^11^ and has also been approved in several Asian countries, including China, Japan, Singapore, and Taiwan.^12^ Subgroup analyses of Asian participants in Clarity AD^13^ and TRAILBLAZER‐ALZ 2^14^ studies (Table 1) demonstrated efficacy and safety findings consistent with those observed in the overall study populations (Supplementary data).

**TABLE 1:** Key clinical outcomes from Asian subgroup analysis of Clarity AD and TRAILBLAZER‐ALZ 2 trials.^13^, ^14^

Parameter	Clarity AD (Lecanemab)	TRAILBLAZER‐ALZ 2 (Donanemab)^‡^
*Subgroup population*	Participants from Japan, Korea and Singapore (*N* = 294)	Participants from Japan (*N* = 88)
*Slowing of clinical progression (%)* *		
CDR‐SB	24.0	–
Global CDR score^†^	–	33.9
*ARIA (%)*		
ARIA‐E	6.2	22.2
ARIA‐H	14.4	26.7
*Infusion‐related reactions (%)*	12.3	6.7

Abbreviations: ARIA‐E, amyloid‐related imaging abnormalities with edema or effusions; ARIA‐H, amyloid‐related imaging abnormalities with cerebral microhemorrhages, cerebral macrohemorrhages, or superficial siderosis; CDR‐SB, Clinical Dementia Rating–Sum of Boxes.

* *Compared to placebo*.

^†^ *Exploratory analysis*.

^‡^ *Combined population (low/medium‐tau and high‐tau)*.

The Asian regional analysis of Clarity AD, which included participants from Japan, Korea, and Singapore, showed a 24% slowing of decline at 18 months and lower incidences of amyloid‐related imaging abnormalities with edema or effusions (ARIA‐E) and ARIA with cerebral microhemorrhages, cerebral macrohemorrhages, or superficial siderosis (ARIA‐H) (Table 1) compared to the incidences in the overall population (12.6% for ARIA‐E; 17.3% for ARIA‐H) with lecanemab.^13^ The incidence of infusion‐related reactions was also lower than in the overall population (26.4%).^13^ Lecanemab was also associated with a relative preservation of health‐related quality of life (HRQoL), with a 23.3% slowing of decline on Quality of Life in AD scale as rated by care partners as proxy, and less increase in caregiver burden (28.7% based on Zarit Burden Interview [ZBI]) in the Asian regional cohort^13^–these findings are similar to those observed in the overall population of Clarity AD.^15^ Initial real‐world experience with lecanemab in approximately 4500 patients in Japan suggested a lower rate of adverse events compared to that reported in Clarity AD^16^; the rates are also consistent with those seen in the Asian regional analysis of Clarity AD.

The Japanese subgroup analysis of TRAILBLAZER‐ALZ 2 reported a 33.9% lower risk of disease progression with donanemab in the combined population based on the global CDR score at week 76, which was consistent with the overall study population.^14^ Donanemab also demonstrated a similar safety profile in the Japanese population (Table 1) to that observed in the overall population.^14^

## 3 COAs AND CLINICAL MEANINGFULNESS–A FOCUS ON LECANEMAB

### 3.1 Clinical endpoints–cognition and function

CDR‐SB is a frequently used primary outcome measure in AD clinical trials–it is a subjective clinician‐reported outcome that combines both cognitive and functional domains and has been validated across different populations.^17^, ^18^ Even small group‐level differences in CDR‐SB (e.g., ∼0.5‐point) may correspond to differences in functional staging; however, such averages do not necessarily imply that all individuals experience clinically meaningful benefit. For example, a 0.5‐point difference can distinguish between “consistent slight or slight benign forgetfulness; partial recollection of events” vs “moderate loss, more marked for recent events, which interferes with daily activities”; or between “slight impairment in community affairs” vs “unable to function independently in these activities”.^17^ In Clarity AD, the adjusted mean group‐level difference in CDR‐SB change from baseline with lecanemab was 0.45 points when compared to placebo at 18 months, reflecting a 27% slowing of disease progression.^2^ This group‐level difference should be interpreted separately from patient‐level changes (e.g., ≥0.5 point), since the mean decline in the placebo group was 1.66.

The 14‐item Alzheimer's Disease Assessment Scale‐Cognitive subscale (ADAS‐Cog14) is a cognitive measure, which is a hybrid of subjective clinician‐reported outcome and performance outcome.^17^ Increases of 2–5 points on the ADAS‐Cog11 or ADAS‐Cog13 in early AD have been shown to reflect clinically significant worsening.^19^ The adjusted mean group‐level difference in the mean ADAS‐Cog14 change from baseline at 18 months with lecanemab was ‐1.44.^2^ Since the thresholds for defining clinically meaningful differences in ADAS‐Cog14 are not yet established, this finding should be interpreted with caution and in the broader context of other functional and global measures.^17^

The Alzheimer's Disease Cooperative Study‐Mild Cognitive Impairment‐Activities of Daily Living scale (ADCS‐MCI‐ADL) is a subjective observer‐reported outcome, modified from ADCS‐ADL to capture changes in instrumental activities of daily living (ADLs) in early AD.^17^, ^20^ A 1‐point change on this scale reflects a difference between performing daily tasks independently vs with supervision, while a 2‐point change distinguishes between performing the tasks independently vs with physical assistance.^17^ In Clarity AD, the adjusted mean group‐level difference in the mean ADCS‐MCI‐ADL change from baseline was 2 points.^2^

### 3.2 Establishing clinical meaningfulness based on clinical endpoints

The concept of minimal clinically important difference (MCID) is based on within‐patient changes, and thus MCIDs cannot be used to determine clinical meaningfulness for group‐level differences.^18^ There is currently no straightforward and standardized method to measure clinical meaningfulness.^17^, ^21^ The European Medicines Agency (EMA) noted the difficulty of interpreting small but statistically significant improvements in group‐level differences seen in AD trials, and recommends performing responder analysis to interpret and establish clinical relevance of the treatment effect,^22^ such as time‐to‐event or time‐saved analyses.

One clinically meaningful outcome is a reduced risk of progression to the next stage of disease. The global CDR score is used to stage AD severity, and the worsening of this score means progression to the next stage of the disease (e.g., from mild cognitive impairment [MCI] to mild dementia or from mild dementia to moderate dementia). Delaying progression to the next global CDR stage represents a clinically meaningful change at patient level since moving from MCI to mild AD or mild AD to moderate dementia impacts the management of subjects with AD, and each stage transition is relevant to subjects, caregivers and clinicians. The time‐to‐worsening analysis on global CDR score demonstrated a 31% lower risk of progression with lecanemab compared to placebo.^2^

The **“**time‐saved” concept is a model‐based, population‐level illustration intended to support communication with patients and caregivers, rather than a directly observed patient‐level outcome. This concept expresses treatment effects in terms of the duration of time by which cognitive or functional loss is delayed (e.g., additional X months to maintain independence with Y months of treatment).^17^, ^18^, ^23^ At the study endpoint of Clarity AD, time‐saved with lecanemab was 5.3 months based on CDR‐SB,^24^ and further slope analysis with extrapolation to 30 months estimated a time‐saving of 7.5 months with lecanemab.^17^

In addition to responder analyses, the inclusion of patient‐ and caregiver‐reported outcomes would support the clinical meaningfulness of the observed clinical outcomes.^17^, ^25^, ^26^ There is currently no validated HRQoL instrument for MCI, but the Quality of Life in Alzheimer's Disease (QOL‐AD), validated for mild to moderate dementia and completed by both the patient and the proxy, is included in AD trials as an exploratory endpoint.^18^ Data from the Clarity AD suggest greater sensitivity of patient‐reported responses to the effects of lecanemab compared with proxy‐reported responses.^15^, ^18^ ZBI, a frequently used instrument to assess caregiver burden, provide additional insights into the clinical meaningfulness of the treatment. Lecanemab benefit was seen across all 22 items of ZBI.^15^

## 4 CLINICAL MEANINGFULNESS FROM THE PERSPECTIVES OF PATIENTS, FAMILIES, AND HEALTHCARE SYSTEMS

**FIGURE 1:**
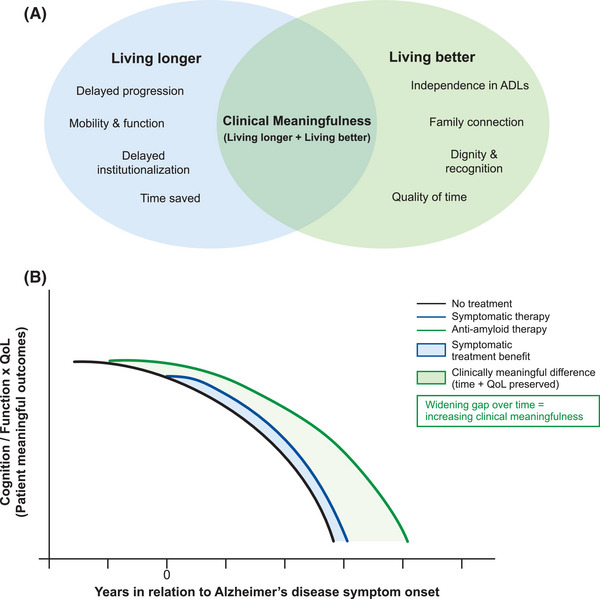
(A) Concept of clinical meaningfulness in Alzheimer's disease. (B) Clinical meaningfulness of AATs in Alzheimer's disease. Compared with symptomatic therapies, AATs not only slow cognitive and functional decline but also preserve meaningful outcomes for patients (cognition, independence, and quality of life). This is reflected in the widening gap between treatment curves over time, representing increasing clinical meaningfulness as each additional year of preserved function delays dependence and institutionalization. In Asia, where family members are often the primary caregivers and healthcare costs are largely borne by families, such benefits translate into reduced caregiver burden and economic impact. AAT, anti‐amyloid therapy; ADL, activities of daily living; QoL, quality of life. Note: The trajectories illustrated in this figure are conceptual averages. In clinical practice, patients experience heterogeneous rates of decline; however, such simplified models are useful for highlighting the potential cumulative impact of therapies over time.

As discussed, primary endpoints in clinical trials mainly focus on cognition and function and may not adequately capture outcomes that are most valuable to patients and caregivers. The concept of clinical meaningfulness goes beyond the statistical significance of trial outcomes and also emphasizes on outcomes that have a tangible impact on patients, caregivers, and healthcare systems. Clinical meaningfulness integrates clinical and QoL domains and aspires to enable patients with AD to live longer with a better quality of life (Figure 1A).

The concept of clinical meaningfulness may be of particular relevance in East and Southeast Asian settings. In countries with rapidly aging societies, such as Japan, Korea, Taiwan, and Singapore, delaying disease progression by even a few months can translate into fewer years of dependency and a substantial reduction in caregiver and healthcare burden (Figure 1B). Equally important, maintaining dignity, staying engaged in community and family life, and minimizing the sense of being a burden to one's children are deeply valued outcomes across many Asian cultures.

Being able to maintain dignity, independence, and perform daily living activities are meaningful outcomes from the patient's perspective.^27^, ^28^ A systematic review by the European Real‐World Outcomes Across the AD Spectrum for Better Care (ROADMAP) consortium revealed that patients, caregivers and healthcare professionals (HCPs) prioritized not only cognitive and functional outcomes assessed in clinical trials, but also the preservation of identity and personality, caregiver burden and access to disease information and healthcare services.^29^ The qualitative study on “What Matters Most” reported that improving memory, modifying disease (halting disease progression or slowing decline) and remaining independent (including the ability to perform daily activities) were treatment outcomes considered valuable to patients across the continuum of AD and their caregivers in the US,^7^, ^8^ which align with the goals of disease‐modifying AAT. Having additional months with independent function due to AAT treatment would also reduce caregiving needs and delay admission into assisted living facilities or nursing homes,^30^ potentially minimizing financial burden. While further cost‐effectiveness studies are needed to fully assess the long‐term impact of AAT on healthcare systems, it can be inferred that delaying disease progression may delay the need for hospitalization, thereby reducing demands on healthcare resources.

## 5 SPECIAL CONSIDERATIONS IMPACTING CLINICAL MEANINGFULNESS IN EAST AND SOUTHEAST ASIA

The findings from the What Matters Most study reinforce the relevance of AAT in early AD treatment, especially in certain healthcare settings where delayed diagnosis and variability in treatment access may amplify the perceived importance of modest clinical gains that extend functional autonomy and preserve dignity. With the rapid transition to a super‐aged society in many Asian countries, the number of older adults living alone is increasing. In this context, the ability to extend the period during which individuals can live independently represents a clinically meaningful benefit of AAT, as it can reduce the need for caregiving and lower the frequency of hospital visits or hospitalization.

Given the benefits of AAT in early AD, early diagnosis is critical but is often delayed.^31^ Informal caregivers are often the first to notice potential symptoms of dementia in individuals with dementia; however, studies involving participants from East and Southeast Asia have identified several barriers to seeking a diagnosis, including lack of knowledge and awareness of dementia (e.g., attributing symptoms to normal aging), emotional denial, resistance from the individuals with dementia, and delays within the healthcare system.^32^, ^33^ Cultural expectations also influence dementia care–in many parts of Asia, caregiving responsibility is placed on family members, and institutional care is considered as a last resort.^32^, ^34^ The proportion of informal care hours is highest in East Asia, accounting for 25.8% of the global hours of informal care.^35^ Public awareness and educational campaigns are therefore imperative to encourage early diagnosis so individuals with AD can receive maximum benefit from treatment and caregiving burden can be reduced.

Genetic differences influence AD manifestation and treatment response in Asian populations. The prevalence of *APOE ε4* allele, an established genetic risk factor for AD, varies across different ethnic groups and geographic regions.^36^ While the proportion of *APOE ε4* carriers is lower in East Asian populations compared to Western populations, the impact of *APOE ε4* on the risk of AD appears to be more pronounced.^36^, ^37^ Importantly, *APOE ε4* is a known risk factor for ARIA, and ARIA occurs more frequently in individuals who are homozygous for *APOE ε4* than in those who are heterozygous.^26^ Nevertheless, data suggests a lower incidence of ARIA events in the Asian regional cohort compared with the global population in the lecanemab clinical trial.^13^ *APOE ε4/4* genotype is also a significant risk factor for cerebral amyloid angiopathy (CAA); it has been associated with severe CAA pathology.^38^ Furthermore, ARIA may be driven by the direct binding of anti‐amyloid antibodies to CAA.^39^ As such, the use of AAT in individuals with preexisting CAA may predispose them to a higher risk of developing ARIA.^38^ White matter hyperintensities, a marker of small vessel cerebrovascular disease, is highly prevalent in many Asian countries and has been associated with worse cognitive performance.^40^ Substantial changes in brain white matter may increase the likelihood of ARIA events.^38^ East Asian patients exhibit a higher risk of intracranial hemorrhage associated with antithrombotic therapies compared with Western patients.^41^ Given the higher cerebrovascular risk in many Asian populations, ARIA‐related risks should be clearly communicated to patients and caregivers during shared decision‐making conversations in the clinic. ARIA risk communication should combine mechanism‐based context (i.e., the roles of *APOE ε4* status, CAA, white matter hyperintensities and concomitant use on antithrombotic therapies in relation to ARIA risk) with practical mitigation strategies (e.g., genotype‐informed monitoring and dosing).

The clinical use of AATs thus necessitates careful monitoring of ARIA, particularly in individuals with higher cerebrovascular risk. The appropriate use recommendations (AURs) for approved lecanemab and donanemab provide recommendations for ARIA detection, reporting and management; yet several practical barriers exist in many Asian countries. Safety magnetic resonance imaging (MRI) scans are recommended during AAT treatment to minimize the likelihood of occurrence or worsening of ARIA. However, MRI infrastructure is limited in many low‐ and middle‐income countries in Asia. Many hospitals in Southeast Asian countries have only one MRI machine available, and there is a recognized need for more MRI systems and trained expertise.^42^ Detection and differentiation of ARIA events from other neurological conditions (e.g., stroke) are critical to preventing misdiagnosis and inappropriate treatment,^43^ underscoring the important role of radiologists in identifying ARIA on brain MRI scans. In addition to ongoing education and training to increase radiologists’ confidence in identifying ARIA, standardized imaging protocol and consistent reporting practices are imperative for ARIA evaluation.^43^ However, given the limited MRI infrastructure in many parts of Asia, maintaining reporting consistency may be challenging, as patients may need to visit different imaging centers. Patients with comorbid vascular diseases (e.g., preexisting microhemorrhages or a history of stroke) who are receiving AAT should have enhanced vigilance for symptoms indicative of ARIA and may therefore require additional MRI scans.

When considering the clinical effectiveness of early AD treatment, unequal access to AAT based on patients’ financial capacity may become a critical issue. Particularly, in middle‐ or low‐income countries in Asia, the high cost of AAT could prevent economically less advantaged individuals from receiving therapy, thereby limiting the overall clinical benefit. As such, cost‐effectiveness data relevant to Asian healthcare systems are needed to support equal access to AAT. Furthermore, inadequate healthcare infrastructure and resource constraints in parts of Asia may also limit clinical use of AAT.

There is a lack of Asian studies evaluating patients’ and caregivers’ perspective on the clinically meaningful impact of treatment benefits on the disease course. Further research in these areas would provide valuable data to inform healthcare policy and support the successful adoption of AAT.

## 6 COMMUNICATING CLINICAL MEANINGFULNESS TO STAKEHOLDERS

Effectively communicating the clinical meaningfulness, associated risks and burdens, as well as available support related to AATs to all stakeholders (i.e., patients and caregivers, clinicians, and policy‐makers) is complex but critical to ensure that individuals with early AD receive maximum benefit from such treatment.

### 6.1 Clinicians

**FIGURE 2:**
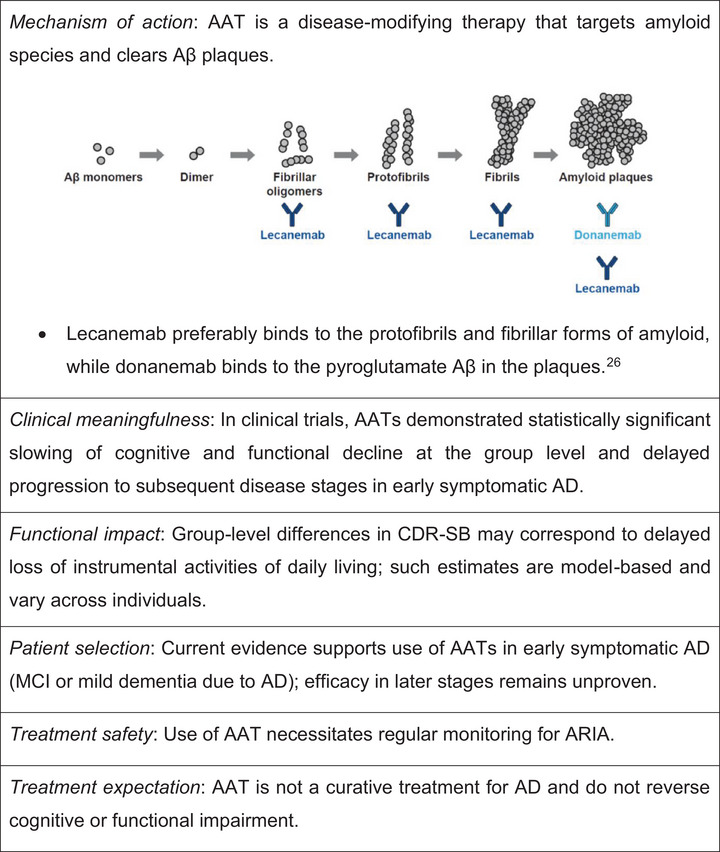
Key points to highlight clinical meaningfulness of AAT to clinicians. AAT, anti‐amyloid therapy; AD, Alzheimer's disease; ARIA, amyloid‐related imaging abnormalities; CDR‐SB, Clinical Dementia Rating–Sum of Boxes; IADL, instrumental activities of daily living; MCI, mild cognitive impairment.

We suggest several key points to effectively communicate the main messages related to AATs to clinicians (Figure 2). Comprehensive education on AATs is undoubtedly mandatory for clinicians intending to manage patients with early AD, and appropriate AURs for approved AATs have been established to ensure their appropriate and safe use.^44^, ^45^

### 6.2 Patients and caregivers

To facilitate meaningful conversations with patients and caregivers about the benefits and limitations of AATs, we propose a novel, structured approach grounded in patient‐centered principles and emerging qualitative research: the Connect, Align, Reframe, and Explain (CARE) communication framework.

The CARE communication framework concept is informed by best practices from the US FDA's Patient‐Focused Drug Development (PFDD) guidance, Alzheimer's Association reports, and communication principles from neurology and dementia care.^46^, ^47^, ^48^ This conceptual framework, which has not undergone formal validation or feasibility testing, has been developed to help clinicians:

- Connect empathetically with patients and caregivers, acknowledging emotional responses to diagnosis and treatment
- Align treatment discussions with individual values and goals
- Reframe complex clinical outcomes (e.g., biomarker changes, CDR‐SB scores) into language that conveys tangible benefits (e.g., preserving independence, delaying loss of function, maintaining hobbies, maintaining driving ability, performing daily activities)
- Explain treatment expectations clearly, including both potential benefits (e.g., slowing decline; time savings; delayed need for full‐time care) and limitations (e.g., risk of ARIA; need for MRI monitoring) to support shared decision‐making.

When communicating with patients and caregivers, using straightforward language and avoiding complex terminology is essential to ensure that they understand the information being shared.^23^ It is helpful to use positive and hopeful language when discussing treatment benefits, but care should be taken to avoid expressions that may create false hope and lead to unrealistic expectations–patients should understand that the goal of AAT treatment is the slowing down of cognitive decline instead of cognitive improvement.^23^, ^49^ Patients and caregivers should be given sufficient time to absorb and digest the information, ask questions, and discuss the benefits, risks, financial costs and logistic burdens associated with the treatment.^23^

**FIGURE 3:**
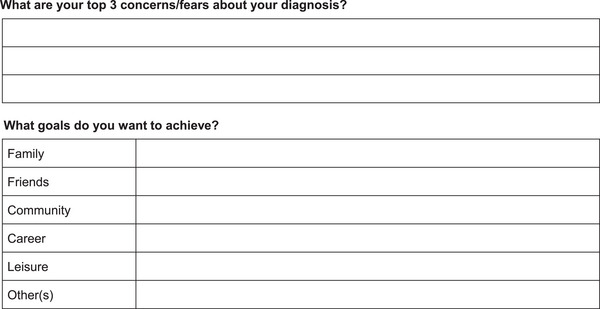
Goal‐setting list.

**FIGURE 4:**
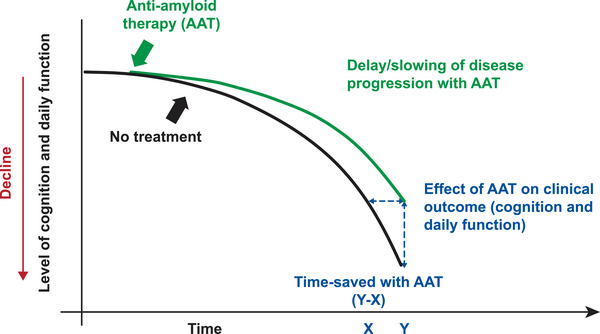
A simple conceptual illustration depicting cognitive and function decline with and without AAT treatment, and “time‐saved” from AAT use. Continuous treatment may increase the magnitude of time‐saved with treatment. AAT, anti‐amyloid therapy. Adapted from Figure 1B, Tarawneh et al., published under the Creative Commons Attribution 4.0 International License (https://creativecommons.org/licenses/by/4.0/).^17^ Note: The magnitude of effect depicted in this figure is not an actual representation of effect observed in clinical trials of AAT. This simplified conceptual figure is intended to support discussion with patients and caregivers about the potential benefits of AAT and the “time‐saved” concept.

**FIGURE 5:**
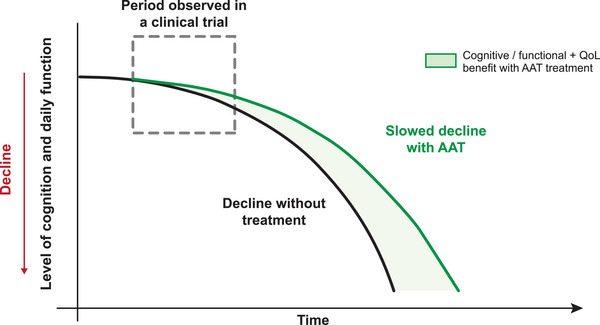
Hypothetical projection of treatment benefits with AAT over time beyond clinical trial period. Without AAT, individuals may decline more rapidly, require increasing caregiver assistance, and transition earlier to assisted living facilities. With AAT, individuals may retain independence, remain at home, and have meaningful interactions with family and friends. AAT, anti‐amyloid therapy; QoL, quality of life. Note: The magnitude of benefit depicted in this figure is not an actual representation of the benefit observed in clinical trials of AAT and is not intended to predict magnitude of benefit of AAT outside of clinical trials. This simplified illustration is intended to support discussion with patients and caregivers about the potential benefits of AAT over time.

We suggest several practical support tools to aid the implementation of the CARE framework:

- *Goal‐setting list*: Connecting with patients and caregivers involves understanding who they are and learning about their fears, hopes and priorities. Creating a goal‐setting list with patients (Figure 3) can provide patients and caregivers with something meaningful to focus on and help align treatment discussion; this can serve as motivation for patients to start or continue treatment.
- *Visual aids*: The use of simple visuals or videos make it easier for patients and caregivers to receive and process information about the disease: how it progresses with and without AAT, how AAT can help delay the disease progression and change disease trajectory, and the potential benefits of AAT in the long term (Figures 4, 5).
- *Easy‐to‐understand Frequently Asked Questions (FAQ) sheet and caregiver resource guide*: Following conversations with patients and caregivers, a FAQ sheet containing key information about the treatment should be given to patients. Additionally, caregivers should receive a resource guide outlining the warning signs of ARIA and the appropriate steps to take if they occur. The guide should also include information about available caregiver support groups.
- *Key motivational messages*: Clinicians may share motivational messages that highlight the clinical meaningfulness of AAT. For example:
- The earlier the treatment with AAT, the better the outcome–treatment benefits can be maximized in the early stages of AD.
- Early treatment with AAT enables you to continue working or being active in society.
- Treatment with AAT may not fully meet your expectations in terms of improving memory, but it offers meaningful benefits–it can help extend the period during which you are able to do your daily tasks independently. During this time, more effective treatments may become available, potentially helping to delay the onset of severe symptoms later in life.
- Testimonials from patients and caregivers: Real‐world stories from patients receiving long‐term treatment and their caregivers, on why they chose to continue treatment and how it impacted their lives, can be a powerful source of encouragement; however, testimonials represent uncontrolled experiential reports and should not be interpreted as evidence of treatment efficacy. Insights from a patient survey conducted at an AD treatment center in the US indicated positive perceptions of lecanemab after long‐term use–patients who stayed on long‐term lecanemab believed the therapy is extending/improving their lives, and all patients who received > 5 years lecanemab were “very satisfied” or “satisfied” with their treatment.^50^ Verbatim testimonials to the question about the most important change observed with ≥5 years lecanemab include:^50^
- Patients: “It saved my life. I would have been a vegetable without it.”, “Stayed stable on treatment, didn't see any changes cognitively.”, “I believe the treatment helped keep my memory stable.”
- Caregivers: “Progression of disease was definitely slowed down.”, “If she had not gotten the treatment, she would have declined much faster.”, “The treatment likely saved her life, and it's hard to know what would have happened without it.”

### 6.3 Policy‐makers

Clinical trial data on AATs have demonstrated statistically significant slowing of disease progression at the group level and favorable QoL outcomes. Policy‐makers should be aware that based on the clinical benefits, institutionalized care for individuals with AD can be delayed thereby saving costs and reducing the burden on the healthcare system. Current data suggest that AATs may not be cost‐effective for healthcare systems in the short term;^51^ however, they have the potential to be cost‐effective in the long term, provided that caregiving needs are reduced or institutionalization is delayed.^52^ Further studies are needed to demonstrate these potential long‐term benefits to the healthcare system. Importantly, there is a need for Asia‐specific cost‐effectiveness studies that account for the diverse healthcare financing and insurance systems across Asian countries.

## 7 LIMITATIONS AND FUTURE DIRECTIONS

The discussion presented in this paper relies primarily on available evidence from Western countries, with limited data from Asia. We acknowledge a general lack of data on Asian populations, including limited representation in AD clinical trials, the absence of outcome measures that are relevant to or adapted to Asian populations, a lack of cost‐effectiveness modelling based on Asian healthcare systems, and a paucity of studies examining patients and caregivers perspectives in the region. As such, the discussion presented serves as a conceptual translation rather than a data‐driven regional validation, pending the availability of more Asia‐specific evidence. The perspectives shared in this paper are limited to the regions represented by the authors and therefore do not reflect the full spectrum of dementia care across Asia. The perspectives also reflect clinical experiences of authors involved in the development and implementation of AATs, which may introduce interpretative bias. We also acknowledge ongoing scientific debate regarding the magnitude, consistency, and long‐term clinical relevance of observed treatment effects of AATs, and that alternative interpretations are valid and continue to evolve.

While AATs demonstrate statistically significant group‐level slowing of decline across multiple clinical endpoints, translating these findings into individual‐level clinical meaningfulness remains complex. Measures, such as CDR‐SB and ADCS‐MCI‐ADL, reflect average treatment effects and may not predict uniform benefit at the individual patient level. Furthermore, disease trajectories in early AD are heterogeneous, and floor and ceiling effects of functional scales may influence perceived benefit in routine clinical practice.

The “time‐saved” concept is presented as a communication aid rather than a direct patient‐level outcome, as it is derived from model‐based extrapolations of group‐level slopes rather than a directly observed patient‐level outcome. Long‐term durability of treatment benefit beyond available trial durations also remains uncertain. These limitations underscore the need for cautious interpretation of data, individualized shared decision‐making, and real‐world longitudinal data to better define sustained clinical meaningfulness.

Lack of knowledge and awareness of dementia remains a barrier to early diagnosis and treatment, highlighting the need for more awareness campaigns and educational programs for patients and caregivers in many parts of Asia. This is particularly important given the maximal benefit gained when initiating AAT at the earliest AD stage.

Greater efforts should be made to enroll Asian participants in all clinical trials for AD drug development. In view of the cultural differences between Asian and Western populations, the development and validation of culturally‐relevant outcome measures or regionally adapted QoL or functional measures (e.g., extended ADLs relevant to different Asian populations) will help generate more accurate data reflective of diverse Asian populations. Additionally, the development of tools to predict treatment response and risk of ARIA will be valuable in clinical practice.

Regular training and educational programs for neurologists, dementia specialists, and radiologists, including cascade‐based safety education models that promote consistent ARIA interpretation and management across centers, play an important role in improving diagnostic accuracy. Regional educational avenues, such as conferences, workshops, or discussion forums, that enable exchange of knowledge across disciplines will facilitate effective detection and management of ARIA. Real‐world data can help refine the safety protocol for AATs and foster discussion among stakeholders in expanding MRI access in local settings.

The accumulation of Asia‐specific real‐world data and the establishment of regional registries will be valuable for monitoring the effectiveness, safety and impact of AATs on patients and their caregivers in the region. Emerging real‐world evidence from China and Japan will help shape and validate treatment practices in other parts of Asia. There is also a need to understand the perspectives of patients and caregivers in Asia regarding what truly matters to them in terms of treatment benefit (e.g., retaining independence in daily tasks, participating in community activities, avoiding becoming a burden on their children). Insights from such studies will encourage the inclusion of relevant Asia‐specific patient‐reported outcomes in future AD trials. Importantly, data from regional registries and country‐specific cost‐effectiveness studies in Asia can help convince policy‐makers to engage in meaningful dialogues with healthcare institutions, pharmaceutical companies, and researchers to improve the affordability and accessibility to AATs and to address health inequities.

Finally, as we gain further understanding of the clinical meaningfulness of AATs, it will be important to implement these insights into practice. Conducting small pilot programs in selected clinics, by incorporating the CARE communication framework concept and support tools into care pathways, can provide validation and demonstrate their feasibility and utility. Data from these pilot programs allow for further refinement before integration into existing national programs (e.g., memory clinics, national dementia strategy), or collaborative care pathways in primary care settings.

## 8 CONCLUSION

AD is a neurodegenerative disease characterized by the progressive accumulation of toxic amyloid species. The recent approvals of AATs signal the beginning of a new chapter in the management of AD. By targeting the underlying disease pathophysiology, lecanemab and donanemab represent important therapeutic developments, demonstrating statistically significant group‐level effects in early symptomatic AD.

Defining and demonstrating clinical meaningfulness of AATs is vital for their successful adoption in clinical practice. This article explores the clinical meaningfulness of AATs and considerations impacting on its meaning in East and Southeast Asia, and how it can be conveyed to patients and caregivers across these regions using the proposed CARE communication framework concept and its practical support tools. Early diagnosis and healthcare system preparedness are key to maximizing the clinical impact of AATs in Asia, and efforts should also be directed toward addressing gaps in these areas.

The adoption of AATs in Asia, however, must go beyond demonstrating clinical efficacy to defining and delivering outcomes that matter to patients, caregivers and healthcare systems. Future efforts should focus on generating independent real‐world evidence, including patient‐reported outcomes; establishing regional registries; conducting region‐specific health economic analyses; and aligning frameworks to capture the full societal value of AAT treatment. Asia, with its diverse healthcare systems and cultural perspectives, is uniquely positioned to shape a more holistic global understanding of clinical meaningfulness in early AD.

## CONFLICT OF INTEREST STATEMENT

Christopher Chen is part of the Eisai global advisory board and Actinogen advisory board; is a site principal investigator for Eisai's AHEAD study and has participated as a site principal investigator in the Clarity AD trial; has received honoraria from Wiley and Medscape for educational webinar and symposium; and serves as President of the Asian Society Against Dementia and as Chairman of the Asian Oceanian Association of Neurologists. Jae‐Hong Lee declares no conflict of interest. Kee Hyung Park has served as an advisory board member for Neurophet; has participated in Eisai and Eli Lilly advisory board meetings; has received honoraria for lectures sponsored by Eisai and Eli Lilly; and has received one‐time consulting fee from Eisai, Eli Lilly and Roche. Chaur‐Jong Hu has served as a roundtable expert for Eisai and Eli Lilly. Vincent Mok declares no conflict of interest. Vorapun Senanarong has received honorarium for lectures from Menarini Thailand, DKSH, Eisai Thailand and Thai Otsuka. Kaori Inaba is an employee of Eisai Co. Ltd. Amitabh Dash is an employee of Eisai (Singapore) Pte Ltd. Author disclosures are available in the supporting information.

## FUNDING INFORMATION

The development of this manuscript was supported by Eisai Co. Ltd., which was limited to the medical writing assistance. Eisai was involved in the preparation of this manuscript.

## Supporting information



Supporting Information

Supporting Information
