# Supplementary material for: Clinical meaningfulness of anti‐amyloid therapies in early Alzheimer's disease: Perspectives from the East and Southeast Asia region
Source: Alzheimers Dement. 2026 Feb 22;22(2):e71230. doi: 10.1002/alz.71230 (PMC12928076; doi:10.1002/alz.71230)
Supplement: Supplementary file 2 — Supporting Information [file ALZ-22-e71230-s001.docx]

**Supplementary data**

The Clarity AD and TRAILBLAZER-ALZ 2 studies have demonstrated significant benefits of lecanemab and donanemab in slowing clinical progression in patients with early AD (**Table**). The TRAILBLAZER-ALZ 2 trial enrolled patients with early symptomatic AD who were amyloid-positive and had either low/medium or high tau pathology as assessed by tau PET.^1^ Patients were stratified by baseline tau levels, and efficacy outcomes were reported for both the low/medium-tau and high-tau groups. In contrast, lecanemab in the Clarity AD trial was studied in a broader early AD population, requiring amyloid positivity but without tau-PET–based inclusion or exclusion criteria, and analyses were conducted across the full enrolled cohort.^2^

**Table**. Key clinical outcomes from phase 3 trials of lecanemab and donanemab^1,2^

|  | **Clarity AD**  (Lecanemab) | **Trailblazer-ALZ 2**  (Donanemab)^‡^ |
| --- | --- | --- |
| ***Slowing of clinical progression (%)**** | | |
| CDR-SB | 27.0 | 28.9 |
| ADCS-ADL-MCI / ADCS-iADL | 37.0 | 27.8 |
| ADAS-Cog | 26.0 | 19.5 |
| Global CDR score^†^ | 31.0 | 37.4 |
| ***ARIA (%)*** | | |
| ARIA-E | 12.6 | 22.4 |
| ARIA-H | 17.3 | 31.4 |

*ADAS-Cog, Alzheimer’s Disease Assessment Scale – cognitive subscale; ADCS-ADL-MCI, Alzheimer’s Disease Cooperative Study – Activities of Daily Living – Mild Cognitive Impairment; ADCS-iADL, Alzheimer’s Disease Cooperative Study – Instrumental Activities of Daily Living; ARIA-E, amyloid-related imaging abnormalities with edema or effusions; ARIA-H, amyloid-related imaging abnormalities with cerebral microhemorrhages, cerebral macrohemorrhages or superficial siderosis; CDR-SB, Clinical Dementia Rating – Sum of Boxes.*

**Compared to placebo.*

^†^*Exploratory analysis.*

^‡^*Combined population (low/medium-tau and high-tau).*

**References**

1. van Dyck CH, Swanson CJ, Aisen P, et al. Lecanemab in Early Alzheimer's Disease. *N Engl J Med*. 2023;388(1):9-21. doi: 10.1056/NEJMoa2212948.
2. Sims JR, Zimmer JA, Evans CD, et al; TRAILBLAZER-ALZ 2 Investigators. Donanemab in Early Symptomatic Alzheimer Disease: The TRAILBLAZER-ALZ 2 Randomized Clinical Trial. *JAMA*. 2023;330(6):512-527. doi: 10.1001/jama.2023.13239.
